# Optical-Based Thickness Measurement of MoO_3_ Nanosheets

**DOI:** 10.3390/nano10071272

**Published:** 2020-06-29

**Authors:** Sergio Puebla, Antonio Mariscal-Jiménez, Rosalía Serna Galán, Carmen Munuera, Andres Castellanos-Gomez

**Affiliations:** 1 Instituto de Ciencia de Materiales de Madrid (ICMM-CSIC), E-28049 Madrid, Spain; sergio.puebla@csic.es (S.P.); cmunuera@icmm.csic.es (C.M.); 2 Laser Processing Group, Instituto de Óptica (IO, CSIC), Serrano 121, 28006 Madrid, Spain; antonio.mariscal@csic.es (A.M.-J.); rosalia.serna@csic.es (R.S.G.)

**Keywords:** MoO_3_, complex oxides, 2D materials, optical microscopy, thickness determination

## Abstract

Considering that two-dimensional (2D) molybdenum trioxide has acquired more attention in the last few years, it is relevant to speed up thickness identification of this material. We provide two fast and non-destructive methods to evaluate the thickness of MoO_3_ flakes on SiO_2_/Si substrates. First, by means of quantitative analysis of the apparent color of the flakes in optical microscopy images, one can make a first approximation of the thickness with an uncertainty of ±3 nm. The second method is based on the fit of optical contrast spectra, acquired with micro-reflectance measurements, to a Fresnel law-based model that provides an accurate measurement of the flake thickness with ±2 nm of uncertainty.

## 1. Introduction

Since the isolation of graphene by mechanical exfoliation in 2004 [1], the catalog of different 2D materials with complementary properties keeps growing [2,3,4,5,6,7,8,9]. Among them, wide bandgap semiconductor materials have attracted a great deal of attention due to their potential in optoelectronic applications [10] requiring electrically conductive materials that are transparent to visible light. Molybdenum trioxide (MoO_3_) in its α-phase is a van der Waals material with a monolayer thickness of ~0.7 nm [11,12] and a direct bandgap of approximately 3 eV [13,14,15], suitable for such applications [16]. It has been used in thin films to enhance the injection of holes in organic light-emitting diodes as a buffer layer [17,18], in organic photovoltaics [19], perovskite solar cells [20] and silicon solar cells [21], furthermore it can be used in gas sensors [15,22,23]. Moreover, MoO_3_ is interesting because of its photochromic, thermochromic, electrochromic effects [24,25,26,27,28] and catalytic properties in the partial oxidation of methanol to formaldehyde [29,30,31,32,33]. Sub-stoichiometric MoO_3_ quantum dots have been synthesized as surface-enhanced Raman scattering substrates [34]. Furthermore, this material displays an in-plane anisotropy of the crystal structure in its layered phase (α-MoO_3_) [16,35,36,37,38], which can be exploited to fabricate novel optical and optoelectronic devices [39,40,41], and anisotropic phonon polariton propagation along the MoO_3_ surface has been observed [42].

A rapid and non-destructive method to measure the thickness of MoO_3_ would be highly desirable for the further development of this line of research. This is precisely the goal of this manuscript: to provide a guide to evaluate the thickness of MoO_3_ nanosheets (in the 0–100 nm range) by optical microscopy-based methods. We propose two complementary approaches: first, a coarse thickness estimation based on the apparent interference color of the flakes and, second, a quantitative analysis of the reflection spectra using a Fresnel law-based model.

## 2. Materials and Methods 

MoO_3_ flakes were grown by a simple physical vapor transport method carried out at atmospheric conditions, developed in Reference [35]. A molybdenum foil was oxidized by heating it up on a hotplate at 540 °C, then a silicon wafer was placed on top. The molybdenum oxide sublimed and crystallized on the surface of the Si wafer, at a slightly lower temperature, forming MoO_3_ flakes. The MoO_3_ grown by this method was characterized by X-ray photoemission spectroscopy (XPS) finding that it was composed of a single-phase fully oxidized MoO_3_ [35]. These MoO_3_ flakes can be easily lifted from the Si wafer surface with a Gel-Film (WF x4 6.0 mil, from Gel-Pak) viscoelastic substrate and subsequently transferred to an arbitrary target substrate. We transferred the flakes onto silicon chips with a 297 nm SiO_2_ capping layer (see the Supporting Information for the quantitative determination of the SiO_2_ thickness) as it is one of the standard substrates for work with 2D materials.

### 2.1. Atomic Force Microscopy (AFM)

AFM characterization was performed at ambient conditions using two commercial AFM systems: (1) a Nanotec AFM system has been used [43] in dynamic mode with a NextTip (NT-SS-II) cantilever (resonance frequency of 75 kHz), (2) an ezAFM (by Nanomagnetics) AFM operated in dynamic mode with Tap190Al-G by Budget Sensors AFM cantilevers (force constant 48 Nm^−1^ and resonance frequency 190 kHz).

### 2.2. Optical Microscopy and Spectroscopy

Optical microscopy images were acquired using a Motic BA310 Me-T microscope (Motic, Barcelona, Spain) (equipped with a 50× 0.55 NA objective and an AMScope MU1803 CMOS Camera) and reflection spectra were collected from a spot of ~1.5–2 µm diameter with a Thorlabs CCS200/M fiber-coupled spectrometer (Thorlabs Inc., Newton, New Jersey, United States). More details about the micro-reflectance setup can be found in Reference [44]. 

## 3. Results and Discussion

**Figure 1:**
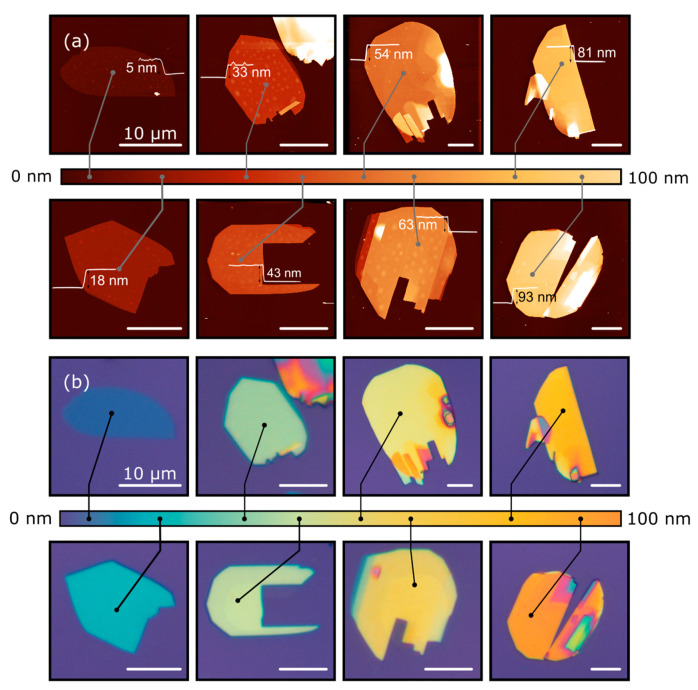
Thickness dependent apparent color of MoO_3_ flakes. (**a**) AFM measurements of the exfoliated flakes with different thickness placed on a 297 nm SiO_2_/Si substrate. (**b**) Optical images of the flakes and a colorbar with the apparent color of flakes with thickness from 5 nm up to 93 nm (from 7 to ~130 layers). Scale bars: 10 µm.

**Figure 2:**
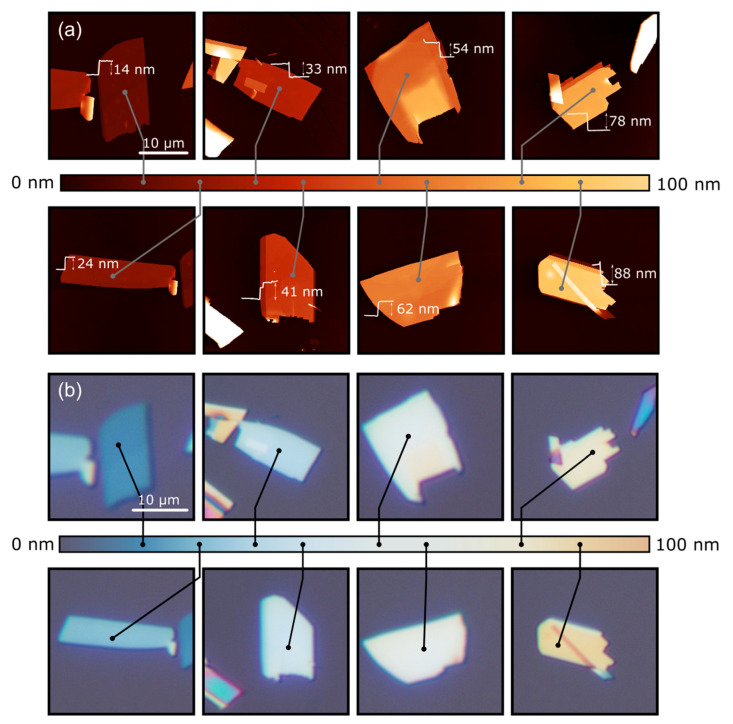
Thickness dependent apparent color of MoO_3_ flakes. (**a**) AFM measurements of the exfoliated flakes with different thickness placed on an 88 nm SiO_2_/Si substrate. (**b**) Optical images of the flakes and a colorbar with the apparent color of flakes with thickness up to 88 nm. Scale bars: 10 µm.

**Figure 3:**
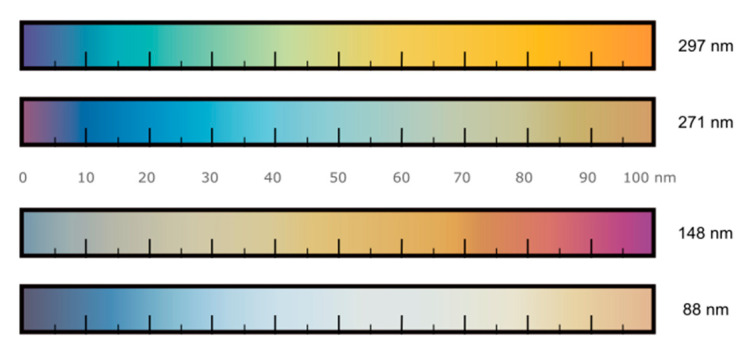
Thickness dependent apparent color of MoO_3_ flakes on SiO_2_/Si substrates with different oxide capping layer thickness.

Figure 1a shows the atomic force microscopy (AFM) topography of eight MoO_3_ flakes with different thicknesses, and Figure 1b shows their corresponding optical microscopy images. The direct comparison between the AFM and the optical images allows us to build up a color-chart correlating the apparent color of the MoO_3_ flakes deposited on top of the 297 nm SiO_2_/Si substrate (the SiO_2_ thickness is experimentally measured by reflectometry with ±0.5 nm uncertainty) with their corresponding thickness with an uncertainty of ±3 nm. Similar approaches have been reported for graphene [45], transition metal dichalcogenides [46], TiS_3_ [47] and franckeite [48]. This method has the main limitation that it requires the use of a specific SiO_2_ thickness as the interference colors of the MoO_3_ flakes strongly depend on the underlying substrate. In this work, we provide color-charts for four different nominal SiO_2_ capping layer thicknesses: 297 nm, 271 nm, 148 nm and 88 nm. Figure 2 shows the correlation of optical images and AFM images for MoO_3_ flakes transferred onto an 88 nm SiO_2_/Si substrate. We address the reader to the Supporting Information for the data corresponding to the 148 nm and 271 nm thick SiO_2_ substrates, named as Figures S1 and S2, respectively. Figure 3 shows a comparison between the apparent color vs. the thickness color-charts obtained for the four different SiO_2_ thicknesses studied here. If a different SiO_2_ thickness is used, a new calibration measurement, like that shown in Figure 1, has to be carried out again for the desired SiO_2_ thickness. 

Further, we quantitatively analyze the reflection spectra of MoO_3_ flakes to measure their thickness more accurately, similarly to previous works in transition metal dichalcogenides, muscovite mica and black phosphorus [44,49,50,51]. Differential reflectance spectra are acquired in normal incidence with a modified metallurgical microscope (BA 310 MET-T, Motic), details in Reference [44]. A spectrum is first acquired onto the bare substrate (*I*_s_) and then onto the flake (*I*_f_) and the optical contrast (*C*) can be calculated as:

(1) $$C=\frac{I_{f}-I_{s}}{I_{f}+I_{s}}$$

**Figure 4:**
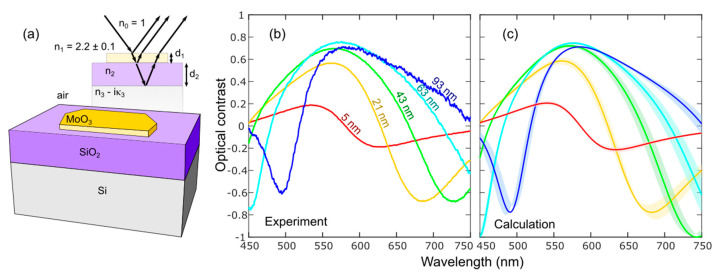
(**a**) Optical model used to calculate the MoO_3_ optical contrast. (**b**) Optical contrast spectra measured on MoO_3_ flakes, on a 297 nm SiO_2_/Si substrate, of different thickness. (**c**) Calculated optical contrast (solid lines) using the Fresnel law-based model [52]. The shaded area accounts for an uncertainty of ±1 nm in the thickness of the flake [53].

In Figure 4a, we sketch the optical system, indicating the different optical media, used to model the optical contrast spectra. The refractive index values of the different media used for the model are shown in Figure S3a. Figure 4b shows the experimental optical contrast obtained for different values of thickness of MoO_3_. 

The optical contrast of this kind of multilayer optical system can be calculated with high accuracy using a Fresnel law-based model [54]. The reflection coefficient in a four media Fresnel model is expressed as [55]:

(2) $$r_{4}=\frac{r_{01}e^{i\left(\phi_{1}+\phi_{2}\right)}+r_{12}e^{-i\left(\phi_{1}-\phi_{2}\right)}+r_{23}e^{-i\left(\phi_{1}+\phi_{2}\right)}+r_{01}r_{12}r_{23}e^{i\left(\phi_{1}-\phi_{2}\right)}}{e^{i\left(\phi_{1}+\phi_{2}\right)}+r_{01}r_{12}e^{-i\left(\phi_{1}-\phi_{2}\right)}+r_{01}r_{23}e^{-i\left(\phi_{1}+\phi_{2}\right)}+r_{12}r_{23}e^{i\left(\phi_{1}-\phi_{2}\right)}}$$

where sub index 0 refers to air, 1 to MoO_3_, 2 to SiO_2_ and 3 to Si. Assuming normal incidence, Φ_i_ = 2πñ_i_d_i_/λ is the phase shift induced by the propagation of the light beam in the media *i*, being ${\tilde{n}}_{i},d_{i}$ and λ the complex refractive index, thickness of the media and wavelength, respectively; r_ij_ = (ñ_i_ − ñ_j_)/(ñ_i_ + ñ_j_) is the Fresnel coefficient at the interface between the media *i* and *j*.

The reflection coefficient in a three media Fresnel model (i.e., the case of the bare substrate without the MoO_3_ flake) is expressed as:

(3) $$r_{3}=\frac{r_{01}+r_{12}e^{-i2\phi_{1}}}{1+r_{01}r_{12}e^{-i2\phi_{1}}}\;$$

where sub index 0 is air, 1 is SiO_2_ and 2 is Si. With these equations, one can calculate the optical contrast by firstly calculating the reflected intensity:

(4) $$R_{k}=\left|\bar{r_{k}}r_{k}\right|\;,\;\forall \;k=3,\;4$$

Then the optical contrast can be defined through the following operation that correlates the reflected intensity by the bare substrate (R_3_) with the reflected intensity by the MoO_3_ flake (R_4_) as:

(5) $$C=\frac{R_{4}-R_{3}}{R_{4}+R_{3}}\;$$

Interestingly, we found that one can accurately reproduce the experimental optical contrast spectra by simply assuming a refractive index of MoO_3_ of ñ_1_ = 2.2 − *i*0. Note that, according to Reference [35], the band structure of MoO_3_ has a negligible thickness dependence and thus we do not expect the refractive index to depend on the flake thickness. The results of the calculated spectra with this model are depicted in Figure 4c. The real part of the refractive index of bulk MoO_3_ n_MoO__3_ spans in the literature from 1.8 to 2.3 [14,56,57]. Since the literature values of the extinction coefficient in the visible range are very low, we neglect that term in our calculations, still providing a good fit to the experimental data. As verification, Figure S3b shows the complex refractive index as a function of wavelength, ñ = *n* + i*κ*, measured with ellipsometry of polycrystalline MoO_3_ films showing values of *n* = 2–2.3 in the visible part of the spectrum (from 1.5 to 3 eV) and a negligible value of *κ* in the same region (from 0.02 to 0.1). This ellipsometry measurement on polycrystalline MoO_3_ verifies that the assumed refractive index for single crystalline MoO_3_ flakes (ñ_1_ = 2.2 − *i*0) is reasonable.

Some of the features present in the optical contrast spectra, like the local maxima and minima, strongly depend on the thickness of the MoO_3_ medium. In fact, the shape of the optical contrast spectra, including the maxima and minima features, arises from the interference colors effect. It is therefore clear that these features will depend on the thickness of the MoO_3_ flake as the optical paths of the light beams passing through flakes with different thicknesses will be different. Therefore, one can evaluate the thickness of a MoO_3_ flake by calculating the optical contrast according to Equations (2)–(5) for different thicknesses of MoO_3_ and determining the best fit by minimum squares.

**Figure 5:**
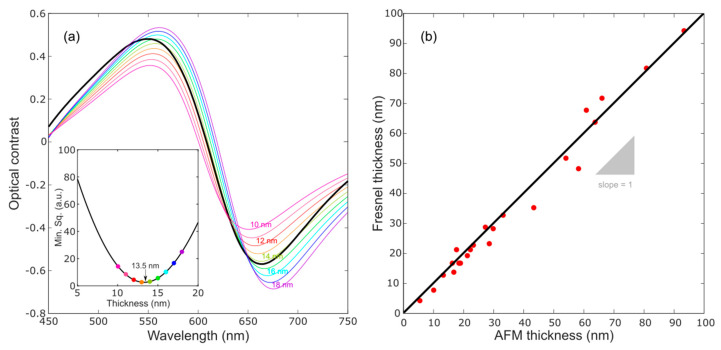
(**a**) Experimental optical contrast (black) and comparison with the calculated ones (color lines) of MoO_3_ flakes, with thicknesses from 10 to 18 nm, placed onto a substrate of 297 nm of SiO_2_. Inset of figure: minimum square value for different values of thickness of the flakes. (**b**) Comparison of thickness measured with AFM and the Fresnel model. Experimental data represented as red dots and line in black has a slope of 1.

Figure 5a compares the optical contrast measured for a MoO_3_ flake (16.5 nm thick according to the AFM) with the optical contrast calculated assuming a thickness in the range of 0–100 nm. The best match is obtained for a thickness of 13.5 nm. The inset in Figure 5a shows the square of the difference between the measured contrast and the calculated one as a function of the thickness assumed for the calculation. The plot shows a well-defined minimum at a thickness of 13.5 nm.

In order to benchmark this thickness determination method, Figure 5b compares the thickness values measured with AFM for 23 flakes from 5 nm to 100 nm thick with the values obtained following the optical contrast fit method discussed above. In the plot, we include a straight line with a slope of 1 that indicates the perfect agreement between the thickness of MoO_3_ nanosheets measured by AFM and the fit to the Fresnel law-based model. The low dispersion along the slope = 1 line indicates the good agreement between the thickness evaluated by both methods. In effect, the calculated linear regression of the data points in Figure 5b has a slope of 1.02.

## 4. Conclusions

In summary, we provided two fast and non-destructive complementary methods to evaluate the number of layers of MoO_3_ nanosheets using optical microscopy. First, one can get a coarse estimation of the thickness (with ±3 nm of uncertainty) by comparing the apparent color of the flakes with a pre-calibrated color-chart. This method is very fast, but it requires the previous calibration of a color-chart that depends on the SiO_2_ capping layer thickness (nonetheless, we provide pre-calibrated color-charts for four commonly used SiO_2_ capping layer thicknesses). The second method is based on the measurement of the optical contrast spectrum of the MoO_3_ flake under study and the subsequent fit to a Fresnel law-based model that includes the optical constants of each medium. This method requires a modification of the optical microscope to allow for differential reflectance measurements, but it can provide lower uncertainty (±2 nm of median standard deviation for the samples on 297 nm of SiO_2_), and it can be easily employed for flakes transferred on SiO_2_/Si substrates with different SiO_2_ thicknesses. We believe that the development of these thickness determination methods can be very helpful for the community working on MoO_3_ as it can effectively speed up the research on this 2D material.

## Supplementary Materials

The following are available online at https://www.mdpi.com/2079-4991/10/7/1272/s1, Figure S1: Color-chart of MoO_3_ on SiO_2_/Si with 148 nm of SiO_2_ capping layer, Figure S2: Color-chart of MoO_3_ on SiO_2_/Si with 271 nm of SiO_2_ capping layer, Figure S3: **(a)** Index of refraction of MoO_3_ films, SiO_2_ and Si materials. These values are obtained from bibliography [56,57,58,59] and they are in accordance with our spectroscopic ellipsometry measurements. **(b)** Index of refraction (n) and extinction coefficient (κ) measured by ellipsometry on a pollycrystalline α-MoO_3_ film transferred onto a SiO_2_/Si substrate with 280 nm of SiO_2_ thickness layer, Figure S4: Calculated optical contrast dependence on illumination wavelength and SiO_2_ thickness for a monolayer MoO_3_ on a SiO_2_/Si substrate.
