# Supplementary material for: Optical-Based Thickness Measurement of MoO3 Nanosheets
Source: Nanomaterials (Basel). 2020 Jun 29;10(7):1272. doi: 10.3390/nano10071272 (PMC7407517; doi:10.3390/nano10071272)
Supplement: Supplementary file 1 [file nanomaterials-10-01272-s001.pdf]

Supplementary Materials:

Optical-Based Thickness Measurement of MoO_3 Nanosheets

Sergio Puebla [1], Antonio Mariscal-Jiménez [2], Rosalía Serna Galán [2], Carmen Munuera [1] and Andres Castellanos-Gomez [1,*]

[1] Instituto de Ciencia de Materiales de Madrid (ICMM-CSIC), E-28049 Madrid, Spain; sergio.puebla@csic.es (S.P.); cmunuera@icmm.csic.es (C.M.)

[2] Laser Processing Group, Instituto de Óptica (IO, CSIC), Serrano 121, 28006 Madrid, Spain; antonio.mariscal@csic.es (A.M.-J.); rosalia.serna@csic.es (R.S.G.)

* Correspondence: andres.castellanos@csic.es

In order to provide more information about the color of MoO_3 flakes deposited on SiO_2/Si substrates, in Figures S1 and S2 are depicted the color-chart that corresponds to 148 nm and 271 nm of SiO_2, respectively. The thicknesses of these flakes (shown at the corner of each image) are calculated using the equations (2)-(5) and are expected to be accurate with approximately ± 1.6 nm of uncertainty, as we have calculated in the previous SiO_2/Si substrates in Figures 1 and 2.

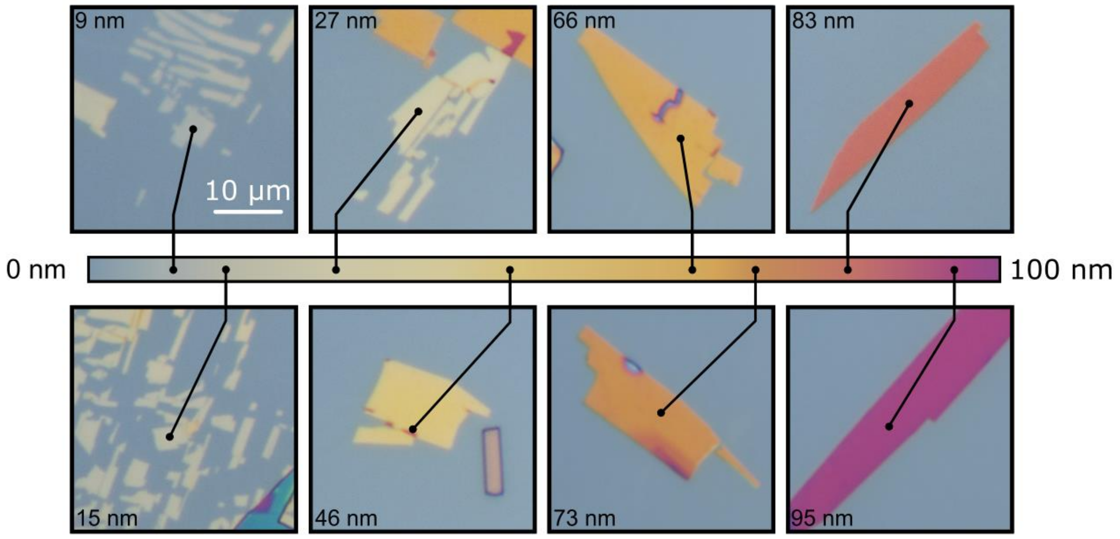

Figure S1: Color-chart of MoO_3 on SiO_2/Si with 148 nm of SiO_2 capping layer.

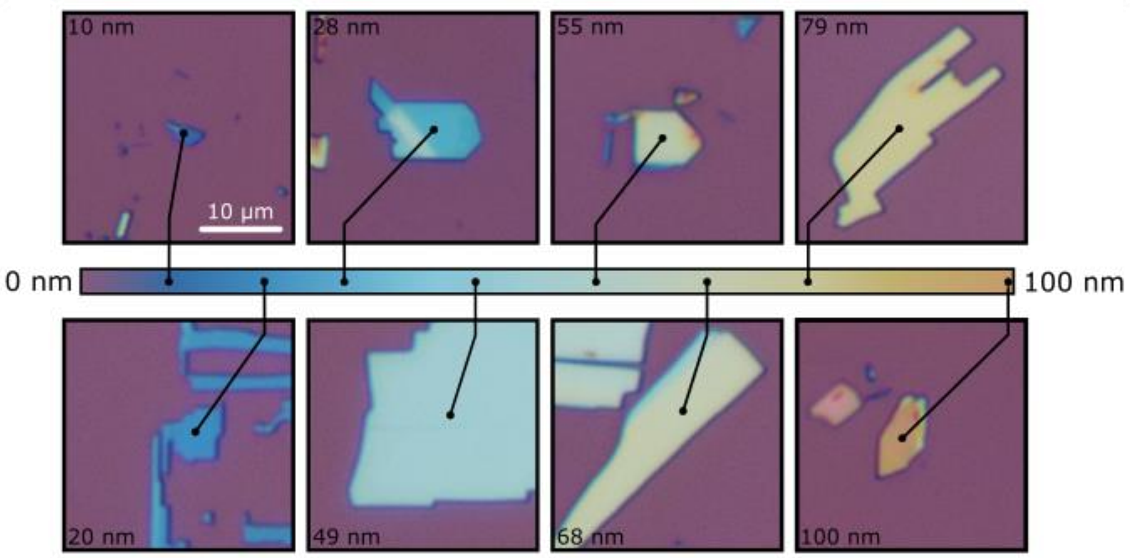

Figure S2: Color-chart of MoO_3 on SiO_2/Si with 271 nm of SiO_2 capping layer.

We have measured, from spectroscopic ellipsometry measurements, the optical constants of continuous α-MoO_3 films grown onto SiO_2/Si substrates with an oxide thickness of 280 nm. For the determination of the complex refractive $\tilde{n}=n+i\kappa$ we have assumed a 4 media model, and we have considered the dispersion values of the refractive index for both Si and SiO_2. The resulting components n and κ as a function of the wavelength are depicted in Figure S3 (b). They have been fitted in the full UV-NIR range (275-1700nm) with a general oscillator model that is a sum of a Tauc-Lorentz oscillator with a bandgap at 3.4 eV, and a Lorentz oscillator for the UV region. Here we show the resulting visible part of the spectrum. It is shown that, in this part of the spectrum, the absorption is negligible ($\kappa < 0.085$), according with the excellent transparency of the flakes in the visible region, and thus it is reasonable to approximate $\kappa = 0$.

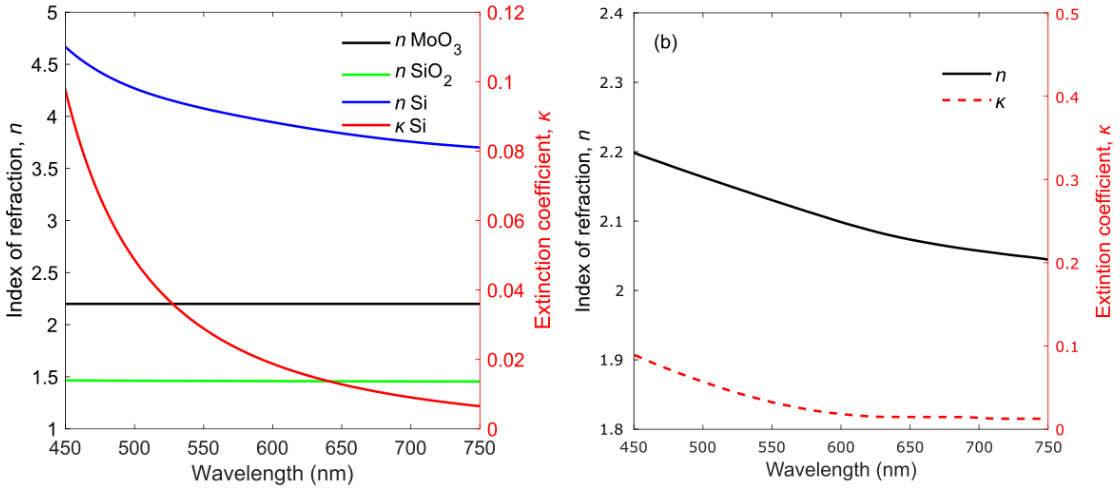

Figure S3: (a) Index of refraction of MoO_3 films, SiO_2 and Si materials. These values are obtained from bibliography [54–57] and they are in accordance with our spectroscopic ellipsometry measurements. **(b)** Index of refraction (n) and extinction coefficient (κ) measured by ellipsometry on a pollycrystalline α-MoO_3 film transferred onto a SiO_2/Si substrate with 280 nm of SiO_2 thickness layer.

Using equations (2)–(5), one can calculate the dependence of the optical contrast for a monolayer MoO_3 flake as a function of the illumination wavelength and the SiO_2 substrate. The results of those calculations are shown in Figure S4. This figure gives the chance to determine the substrate that optimizes its optical identification. For molybdenum trioxide, the SiO_2 thickness values that enhances the optical contrast at a wavelength of 550 nm (where the performance of the human eye is better [59]) are 70, 110, 260 and 300 nm, where the optical contrast is maximum.

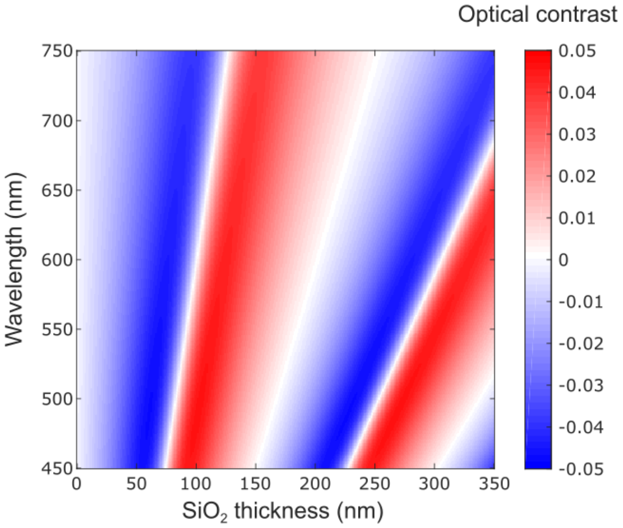

Figure S4: Calculated optical contrast dependence on illumination wavelength and SiO_2 thickness for a monolayer MoO_3 on a SiO_2/Si substrate.
